# A joint transdisciplinary approach for paediatric inflammatory bowel disease: Integrating nursing, dietetic and psychological support

**DOI:** 10.1002/jpr3.70216

**Published:** 2026-07-02

**Authors:** King‐Chi Yau, Laura Tesser, Kay Crook

**Affiliations:** ^1^ Research Department of Clinical, Educational and Health Psychology University College London London UK; ^2^ Paediatric Gastroenterology, St Mark's Hospital London North West University Healthcare NHS Trust London UK

**Keywords:** child, gastroenterology, multidisciplinary

## Abstract

Paediatric inflammatory bowel disease (pIBD) is a long‐term, immune‐mediated condition characterised by fluctuating gastrointestinal symptoms, nutritional risk and psychosocial difficulties. Although multidisciplinary team (MDT) support is considered the gold standard, there is considerable variation in how it is delivered, and it remains common for healthcare services to rely on separate discipline‐specific appointments within the MDT. Here, we describe a transdisciplinary model for pIBD that integrates nursing, dietetic and psychological expertise within a joint appointment. Through the methodology of reflective practice, we identify several potential benefits, including more holistic symptom management, enhanced access to specialist input, reduced stigma around psychological support and improved cross‐disciplinary learning. Potential implementation challenges include implementation feasibility, role ambiguity and demands on some service users. To the best of our knowledge, this paper represents an innovative model of care for pIBD.

## What is Known

- Multidisciplinary team (MDT) care is the recommended standard for paediatric inflammatory bowel disease (pIBD).
- In practice, MDT care is often delivered through separate discipline‐specific appointments or sequential referrals, which can result in fragmented care.

What is New

- This paper describes a joint transdisciplinary clinic model in which nursing, dietetic and psychological care are integrated within a single appointment for children and young people with pIBD.
- Clinician reflections suggest that this approach may support more holistic management, improve access to specialist input, reduce stigma around psychological care and enhance cross‐disciplinary learning.

## 1 INTRODUCTION

Paediatric inflammatory bowel disease (pIBD), primarily comprising Crohn's disease (CD) and ulcerative colitis (UC), is a long‐term, immune‐mediated condition characterised by fluctuating gastrointestinal symptoms, unpredictable flares, nutritional risk and psychosocial challenges.^1^ Children and young people with pIBD often require guidance and support with adhering to treatment, following dietary advice and coping with the physical consequences of the condition, such as fatigue and pain, as well as its psychological impacts, such as distress, low mood and anxiety.^2^, ^3^

### 1.1 Role of the brain–gut axis in inflammatory bowel disease (IBD)

There is increasing recognition of the interconnected nature of these difficulties, particularly through the lens of the brain–gut axis. A recent systematic review and meta‐analysis found that active IBD at baseline significantly predicted later development of mental health difficulties, whilst mental health symptoms at baseline were significantly associated with future risk of flare, hospitalisation and emergency department attendance due to IBD activity.^4^ Emerging research suggests several possible mechanisms, such as alterations in the gut microbiome, through which psychological stress can exacerbate intestinal inflammation.^5^ Conversely, higher disease activity may contribute to greater psychological stress via immune, neural and microbial pathways,^6^ as well as through psychosocial processes such as catastrophising, pIBD‐related uncertainty and shame.^7^ These interactions may be particularly relevant for children and young people, who are navigating key developmental milestones whilst also managing their pIBD, placing them in a more vulnerable state.^7^

### 1.2 Multidisciplinary team (MDT) support

In England, the National Institute for Health and Care Excellence (NICE) produces national evidence‐based guidance and quality standards (QS), which are concise sets of prioritised, evidence‐based statements designed to drive measurable improvements in healthcare quality. NICE QS are frequently referenced internationally. In recognition of the role of the brain–gut axis and the interconnected nature of difficulties associated with pIBD, NICE has adopted a biopsychosocial perspective and published QS81 Quality Statement 2, advocating for MDT support for children and young people with IBD.^8^ A pIBD MDT may include a paediatric gastroenterologist, pIBD specialist nurse, dietitian allocated to paediatric gastroenterology, pharmacist, histopathologist, radiologist and a clinical psychologist with expertise in pIBD. Although MDTs are the gold standard of care for pIBD, there is considerable variation in how MDT support is delivered in IBD,^9^ and it remains common for healthcare services to rely on separate discipline‐specific appointments (e.g., nursing clinic, dietetic appointment, psychological session) or sequential referrals within the MDT (Figure 1). This can result in fragmented care, long waiting times and families needing to repeat their story multiple times.

**Figure 1:**
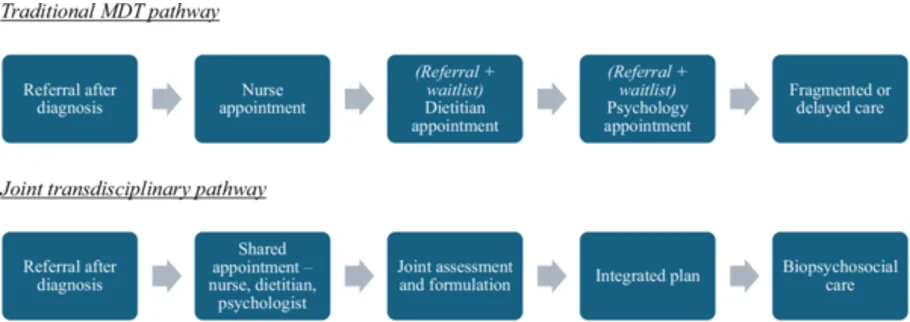
Workflow comparison: Traditional MDT versus joint transdisciplinary approach. MDT, multidisciplinary team.

### 1.3 Transdisciplinary approach

To address these limitations and recognise the interconnected nature of pIBD‐related difficulties, our pIBD team at St Mark's, the National Bowel Hospital, adopts a transdisciplinary approach, in which team members move beyond rigid disciplinary silos, offering input across domains and framing their advice in relation to other aspects of the patient's ongoing care.^10^ In practice, joint transdisciplinary appointments are offered regularly to young patients and their families following a confirmed diagnosis of pIBD. These appointments primarily involve a (i) pIBD advanced nurse, (ii) paediatric dietitian and (iii) clinical psychologist, with all three clinicians seeing young patients and their families together in a shared session. Each team member contributes to a joint biopsychosocial assessment, shared formulation and unified plan. The aim is to deliver holistic, efficient and preventative care whilst normalising dietetic and psychological input as routine components of pIBD medical and holistic management. The current paper aims to (i) describe the pIBD transdisciplinary appointment at St Mark's Hospital, (ii) present a fictional case example and (iii) summarise clinicians' reflections on the perceived benefits.

## 2 METHODS

### 2.1 Ethics statement

Ethics approval was not required for this article, as it describes a novel transdisciplinary model of care and reports clinicians' insights arising from routine reflective practice, rather than involving the collection or analysis of patient‐ or practitioner‐level research data.

### 2.2 Context

Clinicians involved (K. C. Y., L. T. and K. C.) in delivering the joint transdisciplinary clinics engaged in structured reflective practice from 1st September to 28th November 2025, drawing on shared experiences from routine clinical work. Reflections were generated through a combination of written notes following clinics and regular weekly team discussions in which clinicians shared observations from routine clinical practice. These discussions aimed to capture multiple professional perspectives across nursing, dietetics and psychological disciplines.

### 2.3 Statistical analysis

The model of reflective practice was followed.^11^ Reflections were reviewed iteratively to identify recurring patterns and perceived themes related to the delivery of the model. Where differing perspectives arose, these were explored through discussion until a shared understanding was reached, with emphasis placed on identifying areas of consensus whilst acknowledging discipline‐specific viewpoints. These resulting themes informed the organisation of the ‘Perceived potential benefits’ and ‘Perceived potential barriers and limitations’ sections under RESULTS. This approach was intended to capture practice‐based insights rather than constitute formal qualitative research.

## 3 RESULTS

### 3.1 Description of the transdisciplinary appointments

The consultation is delivered as a 30‐ to 60‐min joint appointment with all three core healthcare professionals present, whose main roles include, but are not limited to:

- Paediatric IBD advanced nurse coordinates the consultation, reviews symptoms, monitors medications and guides flare management.
- Paediatric dietitian addresses dietary intake, nutritional risks, growth and feeding challenges.
- Clinical psychologist explores emotional well‐being, procedural anxiety, adherence behaviours, coping strategies as well as individual and familial strengths and resources.

Although the appointment is primarily led by these three healthcare professionals, the paediatric gastroenterologist may join the session when required, especially when diagnostic clarification is needed, when there are significant concerns about disease progression, or when specialist medical guidance is required regarding complex medication decisions. This ensures timely access to specialist advice without requiring separate appointments, and the collaborative, transdisciplinary structure aligns with the chronic, biopsychosocial nature of pIBD, where symptoms, nutritional needs and psychological factors interact continuously over time.^12^ Figure 2 illustrates the shared biopsychosocial formulation and interventions.

**Figure 2:**
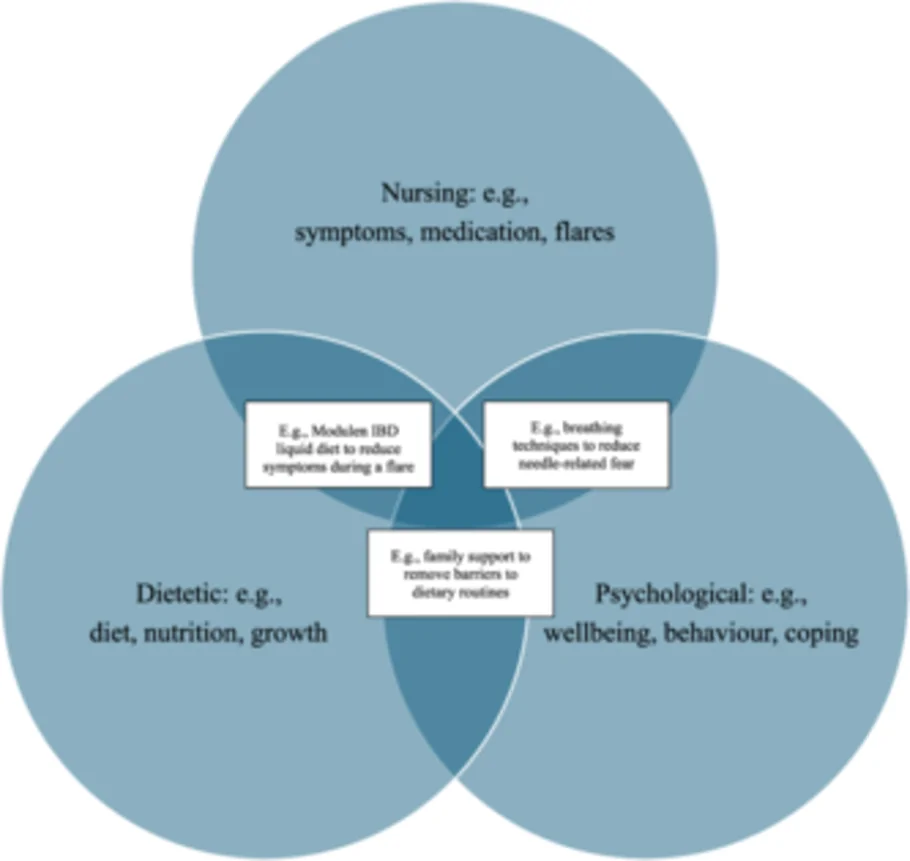
Shared biopsychosocial formulation and interventions. IBD, inflammatory bowel disease.

#### 3.1.1 Indications and timing of transdisciplinary appointments

Joint transdisciplinary appointments are offered regularly within the service and are used flexibly according to clinical need. In our service, children and young people with pIBD are offered an initial joint consultation following diagnosis to support early biopsychosocial assessment and collaborative care planning. Additional joint appointments are arranged selectively when needs span multiple domains, such as during disease flares, nutritional difficulties, treatment adherence concerns or emerging psychological distress. Referral to the joint clinic may be initiated by any member of the pIBD MDT, with clinical judgement guiding whether integrated input is likely to add value. The frequency of follow‐up is individualised, meaning that some patients require only a single joint appointment before transitioning to discipline‐specific follow‐up, whilst others benefit from repeated joint consultations during periods of increased complexity.

#### 3.1.2 A defining feature of the transdisciplinary appointments

A defining feature of the appointment is real‐time shared formulation, created openly in session with all the involved clinicians and the young patient, as well as their family. Working from a biopsychosocial framework in a shared appointment allows interventions to be introduced dynamically and collaboratively. Transdisciplinary interventions may include, but are not limited to, the following. A fictional case example is then presented below to illustrate these potential transdisciplinary strategies.

- Medication and procedural support combined with anxiety‐management techniques.
- Nutritional interventions integrated with behaviour‐change and affect regulation strategies.
- Family‐based approaches to support adherence, routine‐building and overall progress.

### 3.2 Fictional case example

Ari (fictional and fully anonymised), a 15‐year‐old with CD, was referred to the joint transdisciplinary pIBD appointments due to increased abdominal pain and reduced appetite. His parents also described Ari's growing anxiety about blood tests and infusions.

During the shared appointment, the pIBD nurse explored Ari's recent symptoms and medication routines, identifying a decline in adherence to azathioprine. The dietitian noted that Ari was eating irregularly and avoiding many solid foods he associated with pain. The dietitian also learned from his parents that Ari routinely missed breakfast on infliximab infusion days, as he would usually vomit at the start of the infusion. The psychologist explored Ari's beliefs about treatment and diet, during which Ari said, ‘I thought I only need to take the medication when I'm not feeling well’ and ‘Those foods make my tummy hurt’. The psychologist also observed significant anticipatory anxiety around needles after Ari started avoiding certain foods.

Working together, the team developed a shared biopsychosocial formulation linking Ari's difficulties: declining adherence to azathioprine contributed to Ari's abdominal pain, which in turn reduced his appetite. His avoidance of food maintained a heightened state of vigilance, making him more sensitive to perceived threats such as needles. In turn, this fear contributed to Ari's skipping of breakfast on infusion days, creating a vicious cycle. The team co‐created an integrated plan organised around three key transdisciplinary intervention domains:

#### 3.2.1 Medication and procedural support combined with anxiety‐management techniques

To address Ari's misconceptions about treatment, the nurse clarified the purpose of the medicine and the importance of consistent adherence to maintain symptom control and the effectiveness of the infusion. Within the same appointment, the psychologist taught breathing techniques^13^ that Ari could use before and during blood tests and infusions to reduce hypervigilance. This combined approach helped Ari feel more prepared and confident regarding medical treatments and procedures.

#### 3.2.2 Nutritional interventions integrated with behaviour‐change and affect regulation strategies

The dietitian worked with Ari to co‐create a food hierarchy,^14^ organised from easiest to most difficult foods to reintroduce, with the goal of rebuilding a more consistent eating pattern. The psychologist supported this process using behaviour‐change strategies, including motivational interviewing,^15^ to strengthen Ari's confidence and readiness for gradual dietary progression. In addition, the dietitian supported Ari in resuming breakfast regularly, especially on infusion days, by addressing his anticipatory distress around cannulation. During the infusion, the dietitian and psychologist co‐introduced the grounding technique^16^ to help Ari focus on the present moment (e.g., noticing things in the room) rather than anticipating discomfort. Naming the emotions was also introduced to co‐regulate the difficult feelings. As a result, Ari managed not to vomit during the infusion and began to resume eating breakfast afterwards.

#### 3.2.3 Family‐based approaches to support adherence, routine‐building and overall progress

The team collaborated with Ari and his parents to establish predictable routines^17^ around medication and meals. Parents were guided on how to reinforce helpful behaviours, such as taking medication and eating regularly, whilst reducing unintentional reinforcement of avoidance, using principles from operant conditioning. Strengths within Ari (e.g., his willingness to try new strategies) and his family (e.g., openness to guidance) were named and highlighted by all three professionals during the joint session to support ongoing progress and promote self‐management in pIBD.^18^

### 3.3 Perceived potential benefits

Reflecting on the joint transdisciplinary appointments we have delivered for patients with pIBD and their families at St Mark's Hospital, we identified the following potential benefits, supported by relevant literature in pIBD.

#### 3.3.1 More holistic symptom management

Symptoms and difficulties in pIBD rarely fall neatly within one discipline.^12^ Adherence, pain, fatigue and selective eating often involve biological, medical, nutritional and psychological components. The joint transdisciplinary approach potentially allows these interconnected issues to be assessed simultaneously and addressed in a coordinated manner, providing genuine MDT support for children and young people with pIBD.

#### 3.3.2 Improved access and service efficiency

Another perceived benefit of the joint appointment model is the potential to streamline access to specialist input by bringing multiple disciplines together within a single appointment. Barriers to optimal pIBD care have been well documented in the literature.^19^ In the traditional MDT pathway, children may wait weeks or months for separate dietetic or psychological consultations (Figure 1). In some cases, the joint transdisciplinary clinic model may reduce the need for multiple separate appointments or sequential referrals, thereby improving coordination of care. However, we recognise that this approach may also place greater demand on clinical resources, as several professionals are allocated to the same appointment, which could reduce overall clinic capacity and potentially increase waiting times in some settings. The impact on access and waiting times is therefore likely to be context dependent and influenced by local workforce capacity, service organisation and patient complexity. Further service‐level evaluation is required to understand the balance between efficiency gains and resource demands and to identify the moderating variables.

#### 3.3.3 Reduced stigma around psychological support

A systematic review identified ‘perceived social stigma and embarrassment’ as one of the most frequently reported barriers preventing children and young people from seeking mental health support.^20^ Embedding a psychologist within a routine physical health appointment reframes psychological expertise as a core part of pIBD care rather than a separate referral reserved for moderate‐to‐severe mental health difficulties. This normalisation potentially increases engagement, reduces resistance and enables discussions about mood, anxiety, coping and family stressors to occur in a non‐threatening environment.

#### 3.3.4 Cross‐disciplinary professional learning

A significant benefit of the joint transdisciplinary approach has been the reciprocal learning among team members. Working side by side enables clinicians to observe one another's approaches and incorporate relevant techniques into their own routine practice.^21^ For example, our dietitian has developed increased confidence in using grounding technique and emotion‐naming when young patients experienced difficult feelings whilst trying to follow dietary advice; our nurse has integrated breathing technique into routine support for infusion; our psychologist has deepened knowledge of pIBD symptomatology, medication pathways and nutritional considerations. This cross‐disciplinary professional learning enhances the consistency of care across the service, meaning that holistic care extends beyond the joint transdisciplinary appointments.

### 3.4 Perceived potential barriers and limitations

In our experience, potential barriers and limitations of this transdisciplinary model relate primarily to (i) implementation feasibility, (ii) role ambiguity and (iii) demands on some service users. Delivering joint consultations requires coordinated scheduling across multiple professionals and may increase demands on staffing resources, which could present challenges in settings with limited workforce capacity. Furthermore, transdisciplinary working relies on clear role negotiation, shared clinical language and ongoing interprofessional communication to enable the possibility of offering input across professional domains.^10^ Finally, although integrated appointments may normalise psychological and dietetic input, some young people and families may initially feel uncomfortable engaging with multiple clinicians simultaneously, particularly when the disease burden is high.

### 3.5 Contextual considerations and scalability

Whilst this model was developed within a UK tertiary paediatric gastroenterology service with access to specialist nursing, dietetic and psychological expertise, implementation may vary across different healthcare contexts. Workforce availability and local referral pathways are likely to influence the feasibility and scalability of joint transdisciplinary appointments. In particular, non‐tertiary or resource‐constrained settings, as reported in some IBD centres in Australasia, including Australia, New Zealand and Papua New Guinea, may face challenges in accessing specialised nutritional and mental health care, particularly in rural areas.^19^ Conversely, elements of the model may be adaptable through flexible delivery formats, such as partial integration, shared care pathways with other services or virtual participation of specialist professionals. Consideration of local service context will therefore be essential when translating this approach beyond tertiary centres in the UK, and future work should explore how the model can be adapted and evaluated across diverse clinical settings.

## 4 DISCUSSION

To the best of our knowledge, this paper represents an innovative joint transdisciplinary approach integrating nursing, dietetic and psychological support for patients with pIBD. We have outlined the appointment structure and its distinctive features in comparison with the traditional pIBD MDT pathway; presented a fictional case example to demonstrate how transdisciplinary interventions can be implemented in practice; and summarised clinicians' reflections on the perceived benefits and barriers of this model. A key limitation of this paper is the absence of patient‐reported or outcome data to support the benefits described by the involved clinicians. It is recommended that future work could include systematic evaluation, such as a randomised controlled trial (RCT), to further examine the effectiveness of this approach compared with usual care.

## 5 CONCLUSION

Given the innovative nature of this service model and the preliminary observations suggesting improvements in holistic care, access and cross‐disciplinary professional learning, we believe this approach offers a promising and practical addition to the emerging literature on multidisciplinary support within integrated pIBD care.

## CONFLICT OF INTEREST STATEMENT

The authors declare no conflicts of interest.
